# Effects of Agitation, Aeration and Temperature on Production of a Novel Glycoprotein GP-1 by *Streptomyces kanasenisi* ZX01 and Scale-Up Based on Volumetric Oxygen Transfer Coefficient

**DOI:** 10.3390/molecules23010125

**Published:** 2018-01-11

**Authors:** Yong Zhou, Li-Rong Han, Hong-Wei He, Bu Sang, Dai-Lin Yu, Jun-Tao Feng, Xing Zhang

**Affiliations:** 1 Research and Development Center of Biorational Pesticides, Northwest Agriculture & Forestry University, Yangling 712100, Shaanxi, China; zy890619@gmail.com (Y.Z.); hlr4119@126.com (L.-R.H.); hhw920520@163.com (H.-W.H.); fengjt@nwsuaf.edu.cn (J.-T.F.); 2 Shaanxi Research Center of Biopesticide Engineering & Technology, Yangling 712100, Shaanxi, China; 3 Agriculture Research Institute, Tibet Academy of Agricultural and Animal Husbandry Science, Lhasa 850032, Tibet, China; sangbu007@foxmail.com (B.S.); yudailinpz@126.com (D.-L.Y.)

**Keywords:** scale-up, glycoprotein, *Streptomyces*, anti-TMV, volumetric oxygen transfer coefficient

## Abstract

The effects of temperature, agitation and aeration on glycoprotein GP-1 production by *Streptomyces kanasenisi* ZX01 in bench-scale fermentors were systematically investigated. The maximum final GP-1 production was achieved at an agitation speed of 200 rpm, aeration rate of 2.0 vvm and temperature of 30 °C. By using a dynamic gassing out method, the effects of agitation and aeration on volumetric oxygen transfer coefficient (*k*_L_a) were also studied. The values of volumetric oxygen transfer coefficient in the logarithmic phase increased with increase of agitation speed (from 14.53 to 32.82 h^−1^) and aeration rate (from 13.21 to 22.43 h^−1^). In addition, a successful scale-up from bench-scale to pilot-scale was performed based on volumetric oxygen transfer coefficient, resulting in final GP-1 production of 3.92, 4.03, 3.82 and 4.20 mg/L in 5 L, 15 L, 70 L and 500 L fermentors, respectively. These results indicated that constant volumetric oxygen transfer coefficient was appropriate for the scale-up of batch fermentation of glycoprotein GP-1 by *Streptomyces kanasenisi* ZX01, and this scale-up strategy successfully achieved 100-fold scale-up from bench-scale to pilot-scale fermentor.

## 1. Introduction

As an important part of natural products, microbial metabolites exhibit high diversity in producing species, functions, bioactivities and chemical structures, which have gradually become a scientific research hotspot in recent years [1]. *Streptomyces* is a common and well known species of microorganism, and metabolites isolated from *Streptomyces* account for nearly half of the total number of microbial compounds [1,2,3]. Antibiotics and similar small molecule compounds constitute an important group of metabolites produced by *Streptomyces* and usually show various biological activities, representing the main topic of studies and literature all the time [4,5]. With the rapid progress of methods and technologies for screening and isolation, the number of novel microbial metabolites continues to grow, but a number of biological macromolecules, such as polysaccharides, polypeptides and glycoproteins, were also simultaneously discovered [6,7,8,9,10].

It is well known that microbial growth and their metabolite production in bioreactors are greatly influenced by the medium components and physical factors, such as agitation, aeration, temperature, fermentation time and dissolved oxygen (DO) [11]. Both agitation and aeration are important parameters for all aerobic processes, and have a significant effect on the production of most biopolymers, including xanthan [12], gellan [13], pullulan [14], curdlan [15], and so on [16,17,18]. Agitation plays an important mixing and shearing role in fermentation processes. It not only improves mass and oxygen transfer between the different phases, but also maintains homogeneous chemical and physical conditions in the medium by continuous mixing. On the other side, agitation can cause shear forces, which influence microorganisms in several ways, such as changes in morphology, variation in growth and metabolite formation and even causing damage to cell structures. Aeration determines the oxygenation of the fermentation process, and also contributes to mixing of the fermentation broth, especially where mechanical agitation speeds are low [18,19].

Process optimization is generally performed in bench-scale bioreactors, and then scaled up to larger scale for commercial production [20]. The objective of scale-up is to design and build a larger scale system on the basis of the results obtained from small scale devices [21]. The scale-up strategy needs to be based on some criteria such as volumetric oxygen transfer coefficient (*k*_L_a), volumetric power consumption (P/V), impeller tip speed (Vs) and mixing time (t_m_) [22,23,24]. For aerobic fermentation, oxygen supply is one of the key limiting factors for microbial growth and product formation, thus volumetric oxygen transfer coefficient (*k*_L_a) is generally used as a scale-up criterion [25,26]. *k*_L_a plays an important role in the design, scale-up and economy of the fermentation process. This coefficient is considerably influenced by a lot of factors, such as geometrical and operational characteristics of vessels, medium components and the microorganisms’ morphology [27]. A number of methods have been developed for *k*_L_a measurement, such as chemical methods, sodium sulfite oxidation method, absorption of CO_2_, physical methods, and dynamic methods [28,29,30]. The dynamic gassing out method is the most common method and includes the determination of oxygen transfer rate (OTR) and oxygen uptake rate (OUR), respectively [31].

*Streptomyces kanasenisi* ZX01 (CGMCC 4893) was originally isolated from soil around Kanas Lake (Xinjiang Province, China) [32]. Our previous study indicated that strain ZX01 can produce a novel glycoprotein GP-1 with activity against plant viruses, especially tobacco mosaic virus (TMV) [33,34]. However, batch fermentation of strain ZX01 was found to be extremely unstable, affording low GP-1 production and requiring long fermentation periods, which not only reduce tremendously the efficiency of future research, but also seriously hinder potential industrial production of the strain and commercial development of its related fermentation products [35].

In the present work, the effects of agitation, aeration and temperature on the production of glycoprotein GP-1 by *Streptomyces kanasenisi* ZX01 in a 5 L fermentor were investigated systematically. In addition, the process was scaled up from bench-scale (5 L) to pilot-scale (15 L, 70 L and 500 L) on the basis of maintaining the value of *k*_L_a to study the feasibility for future industrial production of glycoprotein GP-1 as a novel anti-plant virus agent.

## 2. Results

### 2.1. Effect of Temperature on the Bench Scale Fermentation of Strain ZX01

**Figure 1:**
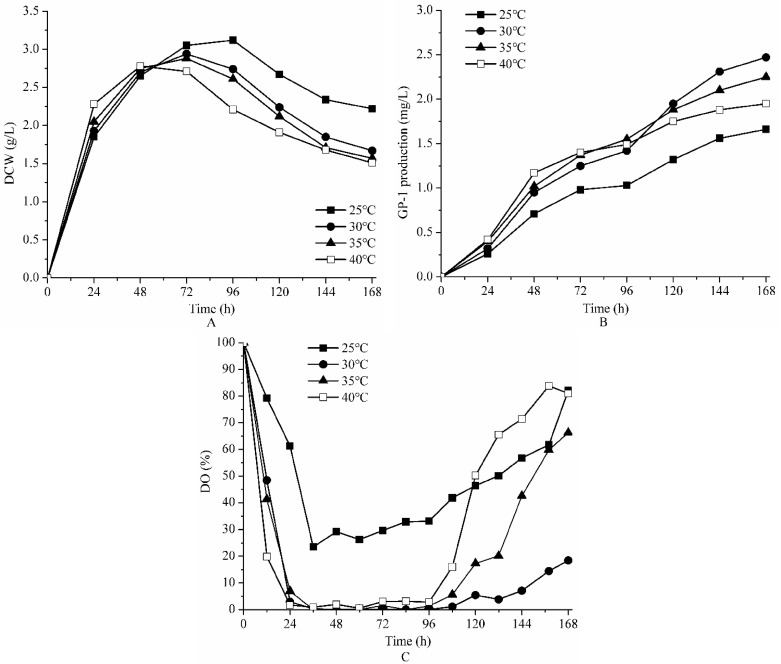
The effect of temperature on DCW (**A**); GP-1 production (**B**) and DO (**C**) during batch fermentation of *Streptomyces kanasenisi* ZX01 in a 5 L fermentor.

Figure 1 shows fermentation results under 25, 30, 35 and 40 °C, with aeration rate and agitation speed controlled at 1.0 vvm and 150 rpm, respectively. As shown in Figure 1A, time course of cell growth was clearly divided into three phases (logarithmic phase, stationary phase and decline phase). The maximum values of dry cell weight (DCW) were 3.15, 2.94, 2.85 and 2.78 g/L at 25, 30, 35 and 40 °C, respectively (Table 1). In other words, a lower temperature was beneficial to growth of strain ZX01 although cell growth rate increased with increase of temperature in the beginning (24 h).

The time course of GP-1 production are illustrated in Figure 1B. In logarithmic phase, GP-1 began to be synthesized and was excreted quickly with cell growth. After a low production rate in stationary phase, GP-1 was largely accumulated in the fermentation broth due to gradual cell death during the decline phase. GP-1 production initially increased with the increase of temperature. After 72 h, the higher temperature of 35 and 40 °C accelerated enzyme inactivation and cell senescence, resulting in a reduction of the GP-1 production rate. The final GP-1 production from 25 to 40 °C were 1.66, 2.47, 2.25 and 1.95 mg/L, respectively (Table 1).

The DO concentration profiles under different temperatures are presented in Figure 1C. All DO concentrations decreased dramatically to a minimum level within 24 h, and then rose slowly during the rest of fermentation time. DO concentrations were so low at 30, 35 and 40 °C, even decreasing to 0% saturation during logarithmic phase and stationary phase. By contrast, DO concentration maintained above 20% saturation during the whole fermentation process at low temperature of 25 °C. That is the reason that GP-1production at 25 °C is much lower than others, because strain ZX01 could not take advantage of oxygen fully to synthesize and produce glycoprotein GP-1. The study indicated that the optimum temperature for *Streptomyces kanasenisi* ZX01 to produce glycoprotein GP-1 was 30 °C.

### 2.2. Effect of Agitation on Fermentation of Strain ZX01 on Bench Scale

**Figure 2:**
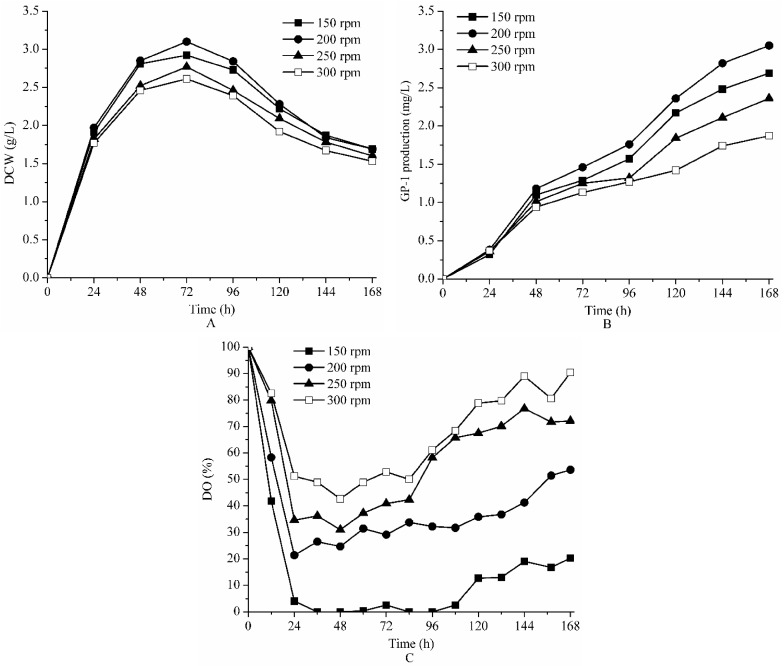
The effect of agitation speed on DCW (**A**); GP-1 production (**B**) and DO (**C**) during batch fermentation of *Streptomyces kanasenisi* ZX01 in 5 L fermentor.

The experiments were conducted at the constant aeration rate of 1.0 vvm and temperature of 30 °C with different agitation speeds of 150, 200, 250 and 300 rpm, respectively (Figure 2).

At first the production of GP-1 increased approximately at the same level under the four agitation speeds, and then became significantly different after 24 h. The maximum final production of GP-1 was achieved at 200 rpm (3.05 mg/L), and the final GP-1 production at 150, 250 and 300 rpm were 2.69, 2.36 and 1.87 mg/L, respectively. The highest values of DCW from 150 to 300 rpm were 2.92, 3.10, 2.77 and 2.61 g/L, respectively (Table 1). The higher agitation speeds of 250 and 300 rpm could cut off mycelium and damage the cell structure owing to unbearable shear force.

The DO concentration profiles were greatly different under four levels of agitation speeds (Figure 2C). DO concentration was distinctly lower at 150 rpm, and maintained below 5% saturation for most of the time. In contrast, DO concentrations were mostly remained above 20%, 30% and 40% saturation at 200, 250 and 300 rpm, respectively. The results showed that 200 rpm was the optimal agitation speed for *Streptomyces kanasenisi* ZX01 to produce glycoprotein GP-1.

### 2.3. Effect of Aeration on Fermentation of Strain ZX01 on Bench Scale

**Figure 3:**
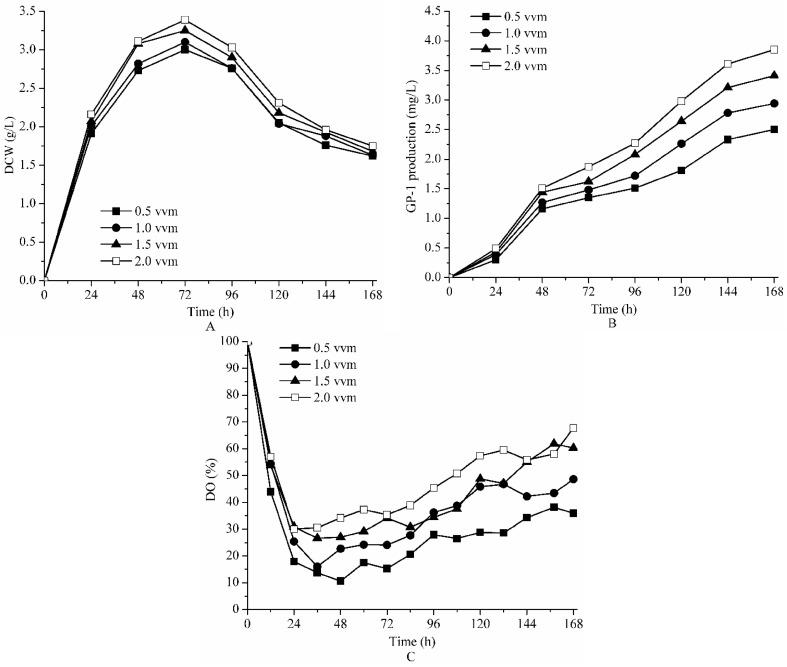
The effect of aeration rate on DCW (**A**); GP-1 production (**B**) and DO (**C**) during batch fermentation of *Streptomyces kanasenisi* ZX01 in a 5 L fermentor.

The fermentations were carried out at different aeration rates of 0.5, 1.0, 1.5 and 2.0 vvm, with same agitation speed of 200 rpm and temperature of 30 °C. Figure 3 shows the effect of aeration rate on GP-1 production, DCW and DO concentration during the fermentation process. In this part of experiment, the final production of GP-1 increased as aeration rate increased (2.50, 2.96, 3.41 and 3.85 mg/L at 0.5, 1.0, 1.5 and 2.0 vvm, respectively) (Table 1). The effect of aeration rate on GP-1 production was more significant than both agitation speed and temperature. On the other hand, the maximum DCW also increased when aeration rate increased (3.00, 3.10, 3.25 and 3.39 g/L at 0.5, 1.0, 1.5 and 2.0 vvm, respectively) (Table 1).

All DO concentration profiles decreased greatly before 24 h, and then remained above 10%, 15%, 25% and 30% saturation at 0.5, 1.0, 1.5 and 2.0 vvm, respectively (Figure 3C). The DO levels in stationary phase increased with increase of aeration rates. The DO concentrations were never less than 10% in all conditions, which indicated that oxygen supply was enough for cell depletion. The result indicated that 2.0 vvm was the optimal aeration rate to obtain a higher production of glycoprotein GP-1.

### 2.4. Effects of Agitation and Aeration on k_L_a on Bench Scale

The volumetric oxygen transfer coefficient (*k*_L_a) represents the capacity of oxygen supply and transfer in the fermentor, which is related with agitation speed, aeration rate, geometrical characteristic of fermentor and rheological character of medium [16]. Effects of agitation speed and aeration rate on *k*_L_a in a 5 L fermentor are shown in Table 1. The *k*_L_a values increased as agitation speed and aeration rate increased on bench scale. At agitation speeds of 150 to 300 rpm, the *k*_L_a values ranged from 14.53 to 32.82 h^−1^. At aeration rates of 0.5 to 2.0 vvm, the values of *k*_L_a were increased from 13.21 to 22.43 h^−1^. The *k*_L_a values had a wider range of change under the effect of agitation speed. The *k*_L_a values were 18.23 and 22.43 h^−1^ at the optimal agitation speed (200 rpm) and aeration rate (2.0 vvm), respectively.

In this study, the final GP-1 production and the maximum DCW decreased when *k*_L_a values increased from 27.21 to 32.82 h^−1^ under the impact of agitation speed (Table 1). In other words, too high *k*_L_a has negative effect on the final GP-1 production and the maximum DCW. The reason should be attributed to tremendous shear force caused by high agitation speed that could destroy the structure of cell and mycelium, and affect the biosynthesis of glycoprotein GP-1.

### 2.5. Bench Scale Verification Experiments

By using one-factor-at-a-time method, temperature, agitation speed and aeration rate on bench scale were sequentially optimized to obtain higher glycoprotein GP-1 production by *Streptomyces kanasenisi* ZX01. Four batches fermentation of *Streptomyces kanasenisi* ZX01 were carried out with 200 rpm, 2.0 vvm and 30 °C to verify the results of optimization experiment. The final GP-1 production, the maximum DCW and *k*_L_a values of verification test are presented in Table 2. The results reveal that under the optimal conditions, the final GP-1 production and the maximum DCW could reach 3.92 mg/L and 3.31 g/L on average. The final GP-1 production of 3.92 mg/L on a bench scale was increased by 54.33% compared with 2.54 mg/L before optimization [35]. On the other side, average value of *k*_L_a was 21.62 h^−1^, which was an important parameter for scale-up fermentation from bench scale to pilot scale.

### 2.6. Scale-Up Fermentation on Pilot Scale

The volumetric oxygen transfer coefficient (*k*_L_a) has been a preferred criterion for scale-up of aerobic fermentations, owing to underlying principle of the oxygen transfer rate in order to achieve a similar oxygen demand for microbial population from a smaller bioreactor to a larger fermentor [25]. Because bench-scale and pilot-scale fermentors have different geometric characteristics and agitation systems, the *k*_L_a values were slightly different despite the fact the same operational conditions were used [36]. A previous experiment with the optimal conditions obtained from the bench scale was tested in pilot-scale fermentors, and both final GP-1 production and *k*_L_a values in 15 L, 70 L and 500 L fermentors were found to be lower than those on bench scale (Table 3).

Therefore, a series of scale-up fermentation experiments with agitation speeds of 225, 250, 275 and 300 rpm were performed in 15 L, 70 L and 500 L fermentors, when temperature and aeration rate were the same as in the bench-scale experiments (Table 3). The results indicated that the highest final GP-1 production found at 225 rpm was 4.03 mg/L with the maximum DCW of 3.54 g/L in 15 L fermentor. As agitation speeds increased from 250 to 300 rpm, the final GP-1 production and the maximum DCW decreased due to the negative effect of shear force. Similar results were also obtained in 70 L and 500 L.

In order to verify the stability of fermentation scale-up process, four batches of repeated experiments were performed in pilot-scale fermentors. The results listed in Table 2 and Table 4 showed that 5 L, 15 L, 70 L and 500 L fermentors achieved final GP-1 production of 3.92, 4.03, 3.82 and 4.20 mg/L, the maximum DCW of 3.46, 3.53, 3.69 and 3.95 g/L, with similar *k*_L_a of 21.62, 22.80, 23.20 and 22.24 h^−1^. Consequently, the batch fermentation of glycoprotein GP-1 by strain ZX01 was successfully scaled up from bench-scale to pilot-scale through controlling agitation speed to maintain *k*_L_a constant. For scale-up of aerobic fermentation, *k*_L_a is frequently used as the criterion to ensure equal oxygen transfer conditions on different scales [25]. The present study also demonstrated that *k*_L_a was applicable to the scale-up of batch fermentation of glycoprotein GP-1 production by *Streptomyces kanasenisi* ZX01 from bench-scale to pilot-scale fermentor.

## 3. Discussion

The research on effects of fermentation conditions such as temperature, agitation and aeration and other parameters during the process, such as dissolved oxygen, pH and reducing sugar on biopolymers production can play an important role in understanding the synthesis of biopolymers, fermentation kinetics and process scale-up [25].

Temperature is one of important factors affecting microbial fermentation, due to its correlation with all biochemical enzyme catalysis processes. Growth and metabolism of microorganisms should proceed under the appropriate temperature. Temperature has various impacts on microbial fermentation, including cell growth, product synthesis, biosynthesis direction and physical properties of broth. According to the kinetics of enzyme catalysis, higher temperatures result in faster reaction rates, leading in turn to sped-up growth and product synthesis. However, when the temperature exceeds the optimum growth temperature of a microorganism, inactivation and denaturation of enzymes will occur, resulting in microorganism death and finally in reduction of the fermentation cycle. Furthermore, the optimum temperatures for cell growth and metabolite accumulation are frequently different. The optimum temperature for growth of glutamic acid-producing strain is 30–34 °C, for example, while the production rate of glutamic acid reaches the maximum in a temperature range of 35–37 °C [37,38,39]. Our results show that the optimum temperature for strain ZX01 growth and GP-1 accumulation were 25 °C and 30 °C, respectively.

Agitation could mainly cause mixing and shear in the fermentation process, which can make oxygen, heat and nutrients mix fully and be transferred efficiently in the fermentation broth, and disperse the air into small bubbles to improve the gas-liquid contact area, and prevent mycelia from clustering to favor of oxygen absorption [19]. Too high an agitation speed not only increases the power consumption, but also creates heterogeneous mixing and shear forces that can damage fragile microorganisms and affect product formation [13]. On the other hand, when the agitation speed is too low, the viscosity of fermentation broth will increase, leading to an reduction in mass transfer efficiency [36]. The research revealed that 200 rpm was the optimal agitation speed for strain ZX01 to grow and produce glycoprotein GP-1.

Aeration not only supplies the necessary oxygen for cell growth, but also eliminates exhaust gas generated during the fermentation process [19]. However, higher aeration rate results in a reduction in the volume of fermentation broth. Oxygen supply is necessary for microorganisms’ growth in aerobic fermentation, but some microorganisms may be affected by oxygen toxicity at excessive oxygen concentration [36]. This oxygen toxicity situation did not occur in our study, since the maximum DCW and final GP-1 production were achieved at the highest aeration rate of 2.0 vvm.

The scale-up of the fermentation process based on constant *k*_L_a for improvement of GP-1 production from bench scale to pilot scale was another important feature in this article, leading to a final GP-1 production of 3.92, 4.03, 3.82 and 4.20 mg/L in 5 L, 15 L, 70 L and 500 L fermenters, respectively. These results indicated that *k*_L_a was quite suitable for the scale-up of batch fermentation process of glycoprotein GP-1 by *Streptomyces kanasenisi* ZX01. In order to ensure equal oxygen transfer conditions on different scales, *k*_L_a has been preferred as the main criterion for scale-up of aerobic fermentation [25]. Kshirsagar et al. reported that PHA production by *Halomonas camposalis* MCMB-1027 was successfully scaled up from 14 L to 120 L by fixing *k*_L_a [40]. Qu et al. achieved a maximum 150-fold scale-up of DHA production from a 10 L to a 1500 L fermentor based on matched *k*_L_a values [20].

There was slight reduction of the final GP-1 production in the 70 L fermentor. In contrast, the result at 500 L was much higher. The hydrodynamic environment in pilot and plant fermenters, including mixing effects, shear forces and oxygen transfer efficiency, is normally more complex than that in bench-scale bioreactors [25]. The increase of broth viscosity caused by high mycelia biomass acts as a barrier making oxygen transfer from the liquid phase to cells difficult [25]. Though agitation speed has a direct effect on mixing that could reduce viscosity and increase oxygen solubility and transfer in broth, shear forces caused by high-speed impeller will have a negative effect on cell growth and metabolite accumulation [41]. More and more researchers have used the Computational Fluid Dynamics (CFD) technique to acquire information about the flow field in fermentors by simulating the fermentor structure. Many of them have reported that the impeller types and combinations have an obviously correlation with the volumetric oxygen transfer coefficient. Homogeneous mixing, moderate shear environment and higher product production could be achieved through optimizing impeller number, types and combinations [42,43,44,45,46]. Hence, in order to improve the flow field and air distribution inside the fermentor, and further increase glycoprotein GP-1 production, different impeller combinations in large-scale fermentor will be optimized by using CFD technique as future work.

In conclusion, our work showed that the maximum glycoprotein GP-1 production by *Streptomyces kanasenisi* ZX01 was obtained at an agitation speed of 200 rpm, aeration rate of 2.0 vvm and temperature of 30 °C in a 5 L fermentor. On the basis of volumetric oxygen transfer coefficient, glycoprotein GP-1 was successfully scaled up from 5 L to 15 L, 70 L and 500 L, resulting in productions of 3.92, 4.03, 3.82 and 4.20 mg/L, respectively.

## 4. Materials and Methods

### 4.1. Microorganism

*Streptomyces kanasenisi* ZX01, obtained from the Research and Development Center of Biorational Pesticides (Yangling, China), was isolated from soil at Kanas Lake (Xinjiang Province, China). Strain ZX01 is registered at the China General Microbiological Culture Collection Center (CGMCC) under strain number CGMCC 4893. The strain was maintained on Gauze’s No. 1 agar medium and subcultured at monthly intervals, or stored in 20% glycerol at −70 °C.

### 4.2. Inoculum Preparation and Media

Strain ZX01 was pre-cultured in Gauze’s No. 1 agar plate for 72 h. After pre-culture, a loop of inoculum from the plate was inoculated into the seed medium of 100 mL for preparation of seed inoculum. For the 5, 15 and 70 L fermentations, seed inoculum was cultured in 500 mL shake flasks with 200 mL working volume at 30 °C and 150 rpm for 72 h. For 500 L fermentation, seed inoculum was cultured in a 50 L seed fermentor containing 17.5 L seed medium at 30 °C and 150 rpm for 72 h.

The seed medium composition was (in g/L): soluble starch, 20; KNO_3_, 1; K_2_HPO_4_, 0.5; MgSO_4_∙7H_2_O, 0.5; NaCl, 0.5; FeSO_4_∙7H_2_O, 0.01. The fermentation medium was (in g/L): millet steep liquor, 10; soluble starch, 10; yeast extract, 3; NaCl, 2.5; CaCO_3_, 0.2.

### 4.3. Fermentors

Four sizes of fermentors were used in this study, and their specifications are listed in Table 5. Bench-scale fermentation was carried out in 5 L quadruple glass bioreactors (GBCN-5C, Zhenjiang East Biotech Equipment and Technology Co., Ltd., Zhenjiang, Zhenjiang, China) with 3.5 L working volume. The bioreactor was equipped with a thermometer, pH sensor, dissolved oxygen sensor, tachometer, air-flow meter and internal pressure sensor and foam-sensing probe. The agitation system consisted of two impellers with four-flat-blade and the magnetic base. The agitation rate was controlled by electromagnetic impulse. The aeration system was an air inlet through a ring sparger with an air-flow meter and filter. Temperature was maintained constant by a heating system in the bottom and cooling water. The bioreactor and all its parts containing 3.5 L medium was sterilized by high pressure steam sterilization pot at 121 °C for 30 min. After sterilization, the fermentation medium was inoculated with 5% (*v*/*v*) seed inoculum. The signal of the foam-sensing probe was connected to an electromagnetic valve through a relay to add antifoam.

Stainless steel fermentors (GUJS-15, GUJS-70 and GJ-500 with nominal 15 L, 70 L and 500 L capacity, and working volumes of 10 L, 50 L and 350 L, respectively, Zhenjiang East Biotech Equipment and Technology Co., Ltd., Zhenjiang, Zhenjiang, China) were used in pilot-scale fermentation. The equipment also included a thermometer, pH sensor, dissolved oxygen sensor, tachometer, air-flow meter and internal pressure sensor and foam-sensing probe. The agitation was provided by two six-flat-blade impellers and driven by mechanical stirred from the top. Air flow was supplied and controlled by spider sparger, air filter and air-flow meter. Temperature was maintained constant by thermostatic water tank and cooling water. Fermentors could be sterilized in-situ with exogenous steam. All data such as DO, pH and temperature were online monitored over the fermentation process and input into a computer at 1 h intervals.

### 4.4. Extraction and Determination of Glycoprotein GP-1 Production

The fermentation broth (100 mL) was centrifuged at 10,000 rpm for 20 min to separate the precipitate and supernatant. The supernatant was concentrated to a volume of 10 mL by rotary evaporator and then precipitated by adding 4-fold volumes of ethanol at 4 °C. The precipitate was redissolved in distilled water (10 mL) and centrifuged (10,000 rpm, 10 min) again to remove those water-insoluble materials. The supernatant was applied to a DEAE-52 Cellulose anion-exchange column (2 cm × 60 cm) eluted with deionized water first, and then with 0.1 M NaCl at a flow rate of 5 mL/min. The 0.1 M NaCl fraction was collected and centrifuged (10,000 rpm, 15 min) with centrifugal filter devices (3 K, 0.5 mL) to remove NaCl. The fraction was subjected to HiTrap^TM^ Con A 4B eluted with binding buffer (20 mM Tris-HCl, 0.5 M NaCl, 1 mM MnCl_2_, 1 mM CaCl_2_, pH 7.4) and elution buffer (0.1 M methyl-α-d-glucoside, 20 mM Tris-HCl, 0.5 M NaCl, pH 7.4) sequentially at a flow rate of 1 mL/min. The fraction eluted with elution buffer that contained GP-1 was concentrated to 100 µL and analyzed by high performance liquid chromatography (HPLC).

The concentration of GP-1 was analyzed by a HPLC (Waters, Milford, MA, USA) with a gel filtration column (TSK-gel G2000SWXL, 7.8 × 300 mm, 5 µm, TOSOH, Tokyo, Japan) and a 996 photodiode array detector at 280 nm. HPLC was performed on 10 µL sample with 20% acetonitrile at a flow rate of 0.5 mL/min and 28 °C. GP-1 purified previously (purity > 99%) was diluted to 10, 5, 2.5, 1.25 and 0.625 mg/mL as standards.

### 4.5. Determination of Dry Cell Weight

The precipitate obtained by centrifuging broth sample was washed with distilled water twice, and then freeze-dried to a constant weight by freeze dryer (SIM International Group Co., Ltd., Norwood, MA, USA), expressed as dry cell weight (DCW, g/L).

### 4.6. Determination of Volumetric Oxygen Transfer Coefficient

OUR, OTR and *k*_L_a were determined by dynamic gassing out method using DO probe [32]. Based on the dynamic mass balance in batch fermentation, the following equation for changes in DO could be established:

(1) $$\frac{{\mathrm{dC}}_{O_{2}}}{\mathrm{dt}}=\mathrm{OTR}-\mathrm{OUR}=k_{L}a\left(C_{O_{2}}^{*}-C_{O_{2}}\right)-Q_{O_{2}}\cdot X$$

where $C_{O_{2}}$ is the dissolved oxygen concentration of the fermentation broth (g/L), $C_{O_{2}}^{*}$ is dissolved oxygen concentration in equilibrium with oxygen partial pressure (g/L), $Q_{O_{2}}$ is specific oxygen uptake rate per unit mass (g/g∙h), X is cell concentration (g/L), and *k*_L_a is volumetric oxygen transfer coefficient (1/h).

The method was divided into two stages, “gas-out” and “gas-in”, which were used to measure OUR and OTR, respectively. In the “gas-out” stage, the inlet of airflow was interrupted, and DO concentration would decrease due to cellular respiration, which could be recorded by DO sensor. OUR was determined by change rate of DO concentration. In the stage, Equation (1) could be simplified to:

(2) $$\frac{{\mathrm{dC}}_{O_{2}}}{\mathrm{dt}}=-\mathrm{OUR}=-Q_{O_{2}}\cdot X$$

The “gas-out” stage must be short and DO concentration should be maintained above the critical oxygen concentration to ensure that OUR was approximately constant and the cell won’t be damaged owing to lack of oxygen.

In “gas-in” stage, the airflow inlet was restarted and DO concentration increased. OTR was determined according to Equation (3):

(3) $$\mathrm{OTR}=\frac{{\mathrm{dC}}_{O_{2}}}{\mathrm{dt}}+\mathrm{OUR}$$

Equation (1) can be rearranged to:

(4) $$C_{O_{2}}=-\frac{1}{k_{L}a}\left(\frac{{\mathrm{dC}}_{O_{2}}}{\mathrm{dt}}+Q_{O_{2}}\cdot X\right)+C_{O_{2}}^{*}$$

Hence, *k*_L_a can be determined by the slope from a plot of $C_{O_{2}}$ vs. ${\mathrm{dC}}_{O_{2}}/\mathrm{dt}+Q_{O_{2}}\cdot X$. The values of *k*_L_a in logarithmic phase (at 12 h) were measured.

### 4.7. Effects of Agitation, Aeration and Temperature on GP-1 Production on Bench Scale

Batch fermentation of strain ZX01 was carried out in 5 L quadruple bioreactors with 3.5 L working volume for 7 days to study the effects of agitation speed, aeration rate and temperature on GP-1 production, DCW and DO concentration. Different agitation speeds were set to 150, 200, 250 and 300 rpm, and aeration rates were adjusted to 0.5, 1.0, 1.5 and 2.0 vvm, while temperatures were controlled at 25, 30, 35 and 40 °C, respectively. Samples were collected from bioreactors at every 24 h interval for measurement of GP-1 production and DCW.

### 4.8. Scale-Up of Fermentation from Bench Scale to Pilot Scale

Batch fermentation on pilot scale was performed in 15 L, 70 L and 500 L fermentors for 7 days. Based on the *k*_L_a value from bench scale, agitation speed and aeration rate on pilot scale were optimized in order to get similar one. Additionally, the production of GP-1 and DCW were measured and compared.

## Figures and Tables

**Table 1:** The final GP-1 production, the maximum DCW and *k*_L_a under different temperatures, agitation speeds and aeration rates in a 5 L fermentor.

Parameters		The Final GP-1 Production (mg/L)	The Maximum DCW (g/L)	*k*_L_a (h^−1^)
Temperature (°C)	25	1.32	3.15	-
	30	2.47	2.94	-
	35	2.20	2.85	-
	40	1.95	2.78	-
Agitation (rpm)	150	2.59	3.02	14.53
	200	3.05	3.12	18.23
	250	2.16	2.67	27.21
	300	1.87	2.61	32.82
Aeration (vvm)	0.5	2.50	3.00	13.21
	1.0	2.96	3.10	16.70
	1.5	3.41	3.25	18.91
	2.0	3.86	3.39	22.43

**Table 2:** The final GP-1 production, the maximum DCW and *k*_L_a in 5 L fermentor.

Fermentor	Fermentation Condition	The Final GP-1 Production (mg/L)	The Maximum DCW (g/L)	*k*_L_a (h^−1^)
5 L	200 rpm, 2.0 vvm	3.89	3.55	22.83
		4.05	3.42	21.31
		3.90	3.41	21.57
		3.83	3.45	20.78
Average		3.92	3.46	21.62

**Table 3:** Measured *k*_L_a values at different fermentation conditions in pilot-scale fermentors.

Fementor	Agitation (rpm)	The Final GP-1 Production (mg/L)	The Maximum DCW (g/L)	*k*_L_a (h^−1^)
15 L	200	3.75	3.55	19.15
	225	4.03	3.54	22.52
	250	3.92	3.40	25.74
	275	3.57	3.20	29.51
	300	3.20	3.14	31.43
70 L	200	3.55	3.60	17.53
	225	3.75	3.60	20.32
	250	3.89	3.76	23.11
	275	3.50	3.58	26.18
	300	3.16	3.32	29.78
500 L	200	3.81	3.77	15.04
	225	3.96	3.81	18.38
	250	4.13	4.01	22.31
	275	4.00	3.85	27.04
	300	3.80	3.66	32.47

**Table 4:** The final GP-1 production, the maximum DCW and *k*_L_a in pilot-scale fermentors.

Fermentors	Fermentation Condition	The Final GP-1 Production (mg/L)	The Maximum DCW (g/L)	*k*_L_a (h^−1^)
15 L	225 rpm, 2.0 vvm	3.98	3.52	23.63
		4.10	3.55	23.71
		4.10	3.61	22.72
		3.94	3.45	21.14
Average		4.03	3.53	22.80
70 L	250 rpm, 2.0 vvm	3.81	3.71	23.42
		3.93	3.76	24.27
		3.78	3.65	23.51
		3.77	3.63	21.59
Average		3.82	3.69	23.20
500 L	250 rpm, 2.0 vvm	4.23	3.93	22.20
		4.28	3.90	23.28
		4.09	4.06	21.81
		4.20	3.91	21.68
Average		4.20	3.95	22.24

**Table 5:** The specifications of fermentors.

Fermentor	Bench-Scale	Pilot-Scale		
Total volume (L)	5	15	70	500
Working volume (L)	3.5	10	50	350
Diameter of fermentor (m)	0.15	0.25	0.38	0.75
Diameter of impeller (m)	0.07	0.10	0.15	0.30
Height of fermentor (m)	0.30	0.50	0.75	1.50
Baffle	3	4	4	6
Impeller	Two impellers with four-flat-blade	Two impellers with six-flat-blade		
Type of drive	Magnetic stirred	Mechanical stirred		
Sterilization	Ex-situ	In-situ		

## References

[1] Berdy J. (2005). Bioactive microbial metabolites. J. Antibiot..

[2] Bibb M.J. (2005). Regulation of secondary metabolism in *Streptomycetes*. Curr. Opin. Microbiol..

[3] Van Wezel G.P., McDowall K.J. (2011). The regulation of the secondary metabolism of *Streptomyces*: New links and experimental advances. Nat. Prod. Rep..

[4] Watve M.G., Tickoo R., Jog M.M., Bhole B.D. (2001). How many antibiotics are produced by the genus *Streptomyces*?. Arch. Microbiol..

[5] De Lima Procópio R.E., da Silva I.R., Martins M.K., de Azevedo J.L., de Araújo J.M. (2012). Antibiotics produced by *Streptomyces*. Braz. J. Infect. Dis..

[6] Berdy J. (1989). The discovery of new bioactive microbial metabolites: Screening and identification. Prog. Ind. Microbiol..

[7] Donadio S., Monciardini P., Alduina R., Mazza P., Chiocchini C., Cavaletti L., Sosio M., Puglia A.M. (2002). Microbial technologies for the discovery of novel bioactive metabolites. J. Biotechnol..

[8] Nwodo U.U., Agunbiade M.O., Green E., Mabinya L.V., Okoh A.I. (2012). A freshwater *Streptomyces*, isolated from tyume river, produces a predominantly extracellular glycoprotein bioflocculant. Int. J. Mol. Sci..

[9] Tong H., Xia F., Feng K., Sun G., Gao X., Sun L., Jiang R., Tian D., Sun X. (2009). Structural characterization and in vitro antitumor activity of a novel polysaccharide isolated from the fruiting bodies of *Pleurotus ostreatus*. Bioresour. Technol..

[10] Gomes R.C., Sêmedo L.T.A.S., Soares R.M.A., Linhares L.F., Ulhoa C.J., Alviano C.S., Coelho R.R.R. (2001). Purification of a thermostable endochitinase from *Streptomyces* RC1071 isolated from a cerrado soil and its antagonism against phytopathogenic fungi. J. Appl. Microbiol..

[11] Potumarthi R., Ch S., Jetty A. (2007). Alkaline protease production by submerged fermentation in stirred tank reactor using *Bacillus licheniformis* NCIM-2042: Effect of aeration and agitation regimes. Biochem. Eng. J..

[12] Borges C.D., Moreira A.D.S., Vendruscolo C., Ayub M.A.Z. (2008). Influence of agitation and aeration in xanthan production by *Xanthomonas campestris* pv pruni strain 101. Rev. Argent. Microbiol..

[13] Giavasis I., Harvey L.M., McNeil B. (2006). The effect of agitation and aeration on the synthesis and molecular weight of gellan in batch cultures of Sphingomonas paucimobilis. Enzym. Microb. Technol..

[14] Wecker A., Onken U. (1991). Influence of dissolved oxygen concentration and shear rate on the production of pullulan by *Aureobasidium pullulans*. Biotechnol. Lett..

[15] Lee I., Kim M., Lee J., Seo W., Jung J., Lee H., Park Y. (1999). Influence of agitation speed on production of curdlan by *Agrobacterium* species. Bioprocess Biosyst. Eng..

[16] Bajaj I.B., Singhal R.S. (2010). Effect of aeration and agitation on synthesis of poly (γ-glutamic acid) in batch cultures of *Bacillus licheniformis* NCIM 2324. Biotechnol. Bioprocess Eng..

[17] Radchenkova N., Vassilev S., Martinov M., Kuncheva M., Panchev I., Vlaev S., Kambourova M. (2014). Optimization of the aeration and agitation speed of *Aeribacillus palidus* 418 exopolysaccharide production and the emulsifying properties of the product. Process Biochem..

[18] Kim S., Hwang H., Xu C., Choi J., Yun J. (2003). Effect of aeration and agitation on the production of mycelial biomass and exopolysaccharides in an enthomopathogenic fungus *Paecilomyces sinclairii*. Lett. Appl. Microbiol..

[19] Mantzouridou F., Roukas T., Kotzekidou P. (2002). Effect of the aeration rate and agitation speed on β-carotene production and morphology of *Blakeslea trispora* in a stirred tank reactor: Mathematical modeling. Biochem. Eng. J..

[20] Qu L., Ren L.-J., Huang H. (2013). Scale-up of docosahexaenoic acid production in fed-batch fermentation by *Schizochytrium* sp. based on volumetric oxygen-transfer coefficient. Biochem. Eng. J..

[21] Schmidt F. (2005). Optimization and scale up of industrial fermentation processes. Appl. Microbiol. Biotechnol..

[22] Rocha-Valadez J.A., Estrada M., Galindo E., Serrano-Carreón L. (2006). From shake flasks to stirred fermentors: Scale-up of an extractive fermentation process for 6-pentyl-α-pyrone production by *Trichoderma harzianum* using volumetric power input. Process Biochem..

[23] Seletzky J.M., Noak U., Fricke J., Welk E., Eberhard W., Knocke C., Büchs J. (2007). Scale-up from shake flasks to fermenters in batch and continuous mode with *Corynebacterium glutamicum* on lactic acid based on oxygen transfer and pH. Biotechnol. Bioeng..

[24] Zou X., Hang H.-F., Chu J., Zhuang Y.-P., Zhang S.-L. (2009). Oxygen uptake rate optimization with nitrogen regulation for erythromycin production and scale-up from 50 L to 372 m^3^ scale. Bioresour. Technol..

[25] Garcia-Ochoa F., Gomez E. (2009). Bioreactor scale-up and oxygen transfer rate in microbial processes: An overview. Biotechnol. Adv..

[26] Huong K.-H., Azuraini M.J., Aziz N.A., Amirul A.-A.A. (2017). Pilot scale production of poly(3-hydroxybutyrate-*co*-4-hydroxybutyrate) biopolymers with high molecular weight and elastomeric properties. J. Biosci. Bioeng..

[27] Galaction A.-I., Cascaval D., Oniscu C., Turnea M. (2004). Prediction of oxygen mass transfer coefficients in stirred bioreactors for bacteria, yeasts and fungus broths. Biochem. Eng. J..

[28] Tobajas M., Garcia-Calvo E. (2000). Comparison of experimental methods for determination of the volumetric mass transfer coefficient in fermentation processes. Heat Mass Transf..

[29] Poughon L., Duchez D., Cornet J., Dussap C. (2003). *k*_L_a determination: Comparative study for a gas mass balance method. Bioprocess Biosyst. Eng..

[30] Moutafchieva D., Popova D., Dimitrova M., Tchaoushev S. (2013). Experimental determination of the volumetric mass transfer coefficient. J. Chem. Technol. Metall..

[31] Tribe L., Briens C., Margaritis A. (1995). Determination of the volumetric mass transfer coefficient (*k*_L_a) using the dynamic “gas out-gas in” method: Analysis of errors caused by dissolved oxygen probes. Biotechnol. Bioeng..

[32] Han L., Zhang G., Miao G., Zhang X., Feng J. (2015). *Streptomyces kanasensis* sp. nov., an antiviral glycoprotein producing actinomycete isolated from forest soil around kanas lake of China. Curr. Microbiol..

[33] Zhang G., Han L., Zhang G., Zhang X., Feng J. (2015). Purification and characterization of a novel glycoprotein from Streptomyces sp. ZX01. Int. J. Biol. Macromol..

[34] Zhang G., Feng J., Han L., Zhang X. (2016). Antiviral activity of glycoprotein GP-1 isolated from *Streptomyces kanasensis* ZX01. Int. J. Biol. Macromol..

[35] Zhou Y., Sun Y.-B., He H.-W., Feng J.-T., Zhang X., Han L.-R. (2017). Optimization of medium compositions to improve a novel glycoprotein production by *Streptomyces kanasenisi* ZX01. AMB Express.

[36] Bandaiphet C., Prasertsan P. (2006). Effect of aeration and agitation rates and scale-up on oxygen transfer coefficient, kLa in exopolysaccharide production from *Enterobacter cloacae* WD7. Carbohydr. Polym..

[37] Redón M., Guillamón J.M., Mas A., Rozès N. (2011). Effect of growth temperature on yeast lipid composition and alcoholic fermentation at low temperature. Eur. Food Res. Technol..

[38] Abdel-Banat B.M., Hoshida H., Ano A., Nonklang S., Akada R. (2010). High-temperature fermentation: How can processes for ethanol production at high temperatures become superior to the traditional process using mesophilic yeast?. Appl. Microbiol. Biotechnol..

[39] Andorrà I., Landi S., Mas A., Esteve-Zarzoso B., Guillamón J.M. (2010). Effect of fermentation temperature on microbial population evolution using culture-independent and dependent techniques. Food Res. Int..

[40] Kshirsagar P.R., Suttar R., Nilegaonkar S.S., Pradhan S., Kanekar P.P. (2013). Scale up production of polyhydroxyalkanoate (PHA) at different aeration, agitation and controlled dissolved oxygen levels in fermenter using *Halomonas campisalis* mcm b-1027. J. Biochem. Technol..

[41] Kamble A., Meena V., Banerjee U. (2010). Effect of agitation and aeration on the production of nitrile hydratase by *Rhodococcus erythropolis* MTCC 1526 in a stirred tank reactor. Lett. Appl. Microbiol..

[42] Zou X., Xia J.-Y., Chu J., Zhuang Y.-P., Zhang S.-L. (2012). Real-time fluid dynamics investigation and physiological response for erythromycin fermentation scale-up from 50 L to 132 m^3^ fermenter. Bioprocess Biosyst. Eng..

[43] Kanda M., Yamamoto E., Hayashi A., Yabutani T., Yamashita M., Honda H. (2010). Scale-up fermentation of echinocandin type antibiotic FR901379. J. Biosci. Bioeng..

[44] Albaek M.O., Gernaey K.V., Hansen M.S., Stocks S.M. (2011). Modeling enzyme production with *Aspergillus oryzae* in pilot scale vessels with different agitation, aeration, and agitator types. Biotechnol. Bioeng..

[45] Duan S., Yuan G., Zhao Y., Ni W., Luo H., Shi Z., Liu F. (2013). Simulation of computational fluid dynamics and comparison of cephalosporin C fermentation performance with different impeller combinations. Korean J. Chem. Eng..

[46] Xia J.-Y., Wang Y.-H., Zhang S.-L., Chen N., Yin P., Zhuang Y.-P., Chu J. (2009). Fluid dynamics investigation of variant impeller combinations by simulation and fermentation experiment. Biochem. Eng. J..

